# Authenticity screening of stained glass windows using optical spectroscopy

**DOI:** 10.1038/srep37726

**Published:** 2016-11-24

**Authors:** Wendy Meulebroeck, Hilde Wouters, Karin Nys, Hugo Thienpont

**Affiliations:** 1 Vrije Universiteit Brussel, Applied Physics and Photonics Department, Brussels Photonics Team B-PHOT, Brussels, 1050, Belgium; 2 Vrije Universiteit Brussel, Department of Art Studies and Archaeology, Brussels, 1050, Belgium

## Abstract

Civilized societies should safeguard their heritage as it plays an important role in community building. Moreover, past technologies often inspire new technology. Authenticity is besides conservation and restoration a key aspect in preserving our past, for example in museums when exposing showpieces. The classification of being authentic relies on an interdisciplinary approach integrating art historical and archaeological research complemented with applied research. In recent decades analytical dating tools are based on determining the raw materials used. However, the traditional applied non-portable, chemical techniques are destructive and time-consuming. Since museums oftentimes only consent to research actions which are completely non-destructive, optical spectroscopy might offer a solution. As a case-study we apply this technique on two stained glass panels for which the 14^th^ century dating is nowadays questioned. With this research we were able to identify how simultaneous mapping of spectral signatures measured with a low cost optical spectrum analyser unveils information regarding the production period. The significance of this research extends beyond the re-dating of these panels to the 19^th^ century as it provides an instant tool enabling immediate answering authenticity questions during the conservation process of stained glass, thereby providing the necessary data for solving deontological questions about heritage preservation.

Window glass composition changes throughout time^1^. Major compositional groups within the wood and plant tradition (~800–1800 AD) are defined based on differences in calcium, potassium and sodium concentration and include potash, high lime low alkali (HLLA) and mixed-alkali glass^2^. Medieval glasses in the Low Countries typically have a potash composition. At the end of the 15^th^ century the potash type was gradually abandoned in favour of the HLLA glass. From the end of the 17^th^ century to the early 19^th^ century, most window glass is mixed-alkali^3^. Subsequently in the 19^th^ century industrial produced soda is used for glass manufacturing^4^,^5^.

Stained glass windows typically hold an assemblage of coloured pieces. Depending on the production phase, the individual panes are classified as being naturally-coloured, pot-coloured or flashed. Unintentionally (natural) colouring arrives from metal impurities in the raw materials. Intentionally colouring is imposed by adding metal oxides; pot-coloured glass refers to body-tinted glass while flashed glass is composed of a clear glass flashed with one or multiple thin coloured glass layers.

On top of the glass decoration layers based on painted details in combination with yellow stain are often applied to enhance the design. The use of silver stain is omnipresent in the 14^th^ century and became generally applied in the 15^th^ and 16^th^ century^6^. The procedure consists of coating the glass surface with a silver compound dispersed in a clay medium and firing at a temperature just above the glass transition temperature. During this process silver ions are exchanged with the alkali ions from the glass (Na+ or K+) and diffuse in the glass. Subsequently they reduce to the metallic state with the growth of silver nanoparticles. The diffusion coefficients and the redox reactions depend on the composition of the glass. In addition, also the production parameters (firing temperature and time) as well as the composition of the coating play an important role in the staining process. The colour depends on the size, shape and concentration^7^ and is the result of localized absorption and scattering. The recipe of silver stain was first described by Antoine de Pise, only using silver^8^. In the late 15^th^ century the interest to obtain brighter yellow and orange colours grew and the combined use of silver and copper was exploited to increase the size of the colloids and broaden the colour range^6^.

The above described chronological differences in glass composition, make that authenticity questions of stained glasses often go hand-in-hand with the study of the window’s composition. In recent years attention focused on non-destructive and portable analysis methods for measuring glass composition. This as a replacement for the electron- or X-ray based analytical techniques such as Wavelength-Dispersive X-ray spectrometry (SEM-WDS), Electron Probe Micro-Analysis (EPMA), or Laser Ablation Inductively Coupled Plasma Mass Spectrometry (LA-ICP-MS) which require expensive laboratory equipment and sample pre-treatment. Technological developments led to the use of handheld X-Ray-Fluorescence (XRF) equipment^9^,^10^, mobile instruments for Raman analysis^11^,^12^ and portable spectrophotometers^13^. Our team has proven the advantages of applying UV-VIS-NIR spectroscopy as a first-line analytical technique. It is fast and relatively cheap, essentially non-destructive and it can be used *in-situ* by trained art historians and archaeologists^13^,^14^,^15^,^16^. Since the colouring agent signature depends on the composition of the glass, optical absorption spectroscopy can be used as a first-line technique to unveil information about the glass composition of coloured glasses. The purpose is to relate a specific glass composition to a characteristic spectral pattern consisting of one or several absorption bands located at well-defined spectral wavelengths. These absorption bands are the spectral fingerprint of the transition metals. So far all our cases have been limited to the study of naturally-coloured or pot-coloured glasses. The use of UV-VIS-NIR spectroscopy as archaeometric analysis tool has already been applied to identify the pigments in several other types of ancient materials such as ceramics and glazes^17^,^18^, paintings^19^, paper and ink artefacts^20^ as well as stone monuments and wall paintings^21^.

The objective of this paper is to verify if UV-VIS-NIR spectroscopy can be used to give a conclusive answer when dating stained windows. Therefore, we need to extend our domain of expertise from the study of pot-coloured and naturally-coloured glasses to spectral research on flashed and stained glasses. As a case-study we investigate two remarkable intact stained glass panels in the permanent exhibition of the museum ‘Ten Duinen 1138’, which is located on the ruins of the former medieval Cistercian Abbey of the Dunes at Koksijde (Belgium). The two panels are believed to be authentic architectural canopies from two different windows of the former abbey that was founded in the 12^th^ century and abandoned around 1600. In the early 1960’s both panels were dated to the 14^th^ century by the art historian Jean Helbig^22^. His conclusions were solely based on stylistic similarities with the windows of the church Saint-Ouen in Rouen (France) dated to 1325; and the windows of Klosterneuburg Monastery (Austria) dated around 1330. However, as it is commonly known that ancient motifs were frequently copied in later periods, it is not surprising that archival research we conducted unveiled several neo-gothic (19^th^ – early 20^th^ c.) windows with similar stylistic characteristics. Two additional visual observations strengthen the questioning of a 14^th^ century origin of the panels: (1) The glass is physically very complete and the environment has not taken its toll over centuries. A detailed survey highlights a complete absence of any weathering of the glass or painted surfaces. If the glass would be 14^th^ century material, then the condition of the potash rich glass would have been highly deteriorated displaying extensive traces of corrosion^23^. (2) The lead cames are not comparable to 14^th^ century material. The flanges of the cames demonstrate characteristic regular-shaped cross-sections and the core of all lead cames show clear and straight parallel tooth marks both coming from the milling process^24^. Although the exact date of invention of the mill remains unclear, written sources hint at the later 15–16^th^ century.

Since none of the mentioned macroscopic observations is fully conclusive, we further investigate all 85 panes of the two glass panels by optical spectroscopy. The methodology is to discover the fabrication date by unravelling the applied glass composition via the study of the spectral properties of the different groups; i.e. pot-coloured, flashed, naturally-coloured and stained glass. The research is divided in two parts. A first focus is put on flat glass as a canvas. For the pot- and naturally-coloured fragments emphasis is put on the study of the metal oxide absorption bands. For the flashed glasses we analyse the nanoparticle properties (type and dimensions) and compare them with the properties of a collection of historic flashed samples for which a similar metal was applied for colouring. The selected samples cover a range of different glass compositions. For the investigation of the decoration layers we follow an analogue approach; we investigate the spectral properties of the silver stained pieces and verify if the spectral properties match with those of well-dated historic samples described in literature.

## Results

### Setup of the panels

**Figure 1:**
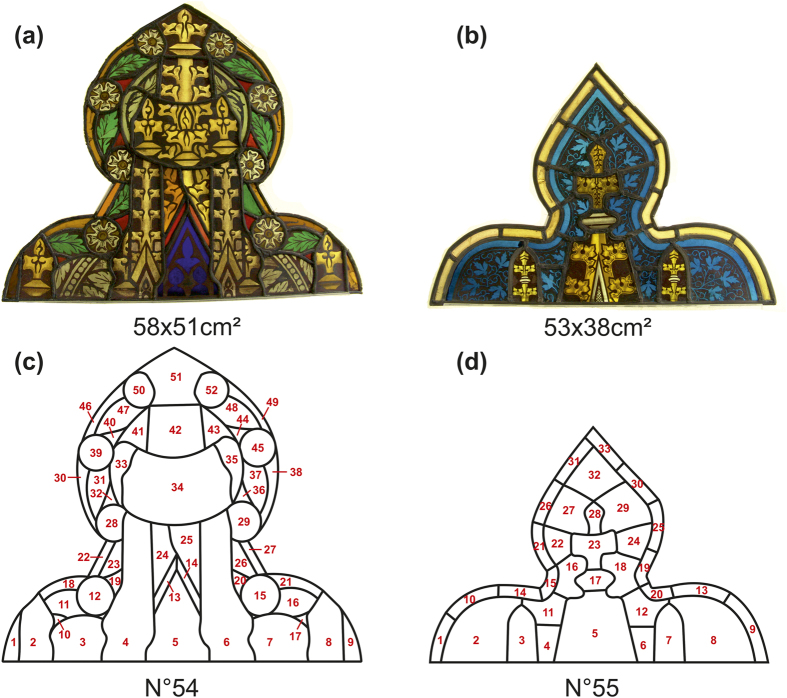
(**a**,**c**) Panel N°54 contains 52 panes and is painted in brown-reddish vitreous paint on naturally coloured glass combined with dominant silver stain. The clear strong colours surrounding the central canopy are striking, using blue, green and yellow pot-coloured and red flashed glass. (**b**,**d**) Panel N°55 contains 33 panes and is painted with a black vitreous paint on naturally coloured glass with details picked out in yellow stain. The central motif is surrounded by blue pot-coloured decorating fragments bordered with plain white-yellowish glass. (© Abdijmuseum Ten Duinen, Gemeentebestuur Koksijde).

The panels (Fig. 1) show architectural canopies from two different windows, both designed in complete symmetry. For four panes it is known that they were restored (54–9, 54–22, 54–31 and 55–4).

### Technical examination by optical spectroscopy

As a first step all measured spectra are classified in separate groups based on the observed spectral shapes. In the coming part of the paper we will refer to the spectral groups rather than to the pane numbers in order not to overload the text. Supplementary Table 1 summarizes the spectral features of each individual pane including the CIE1931 colour value (see paragraph on methods).

### Intentionally pot-coloured panes

**Figure 2:**
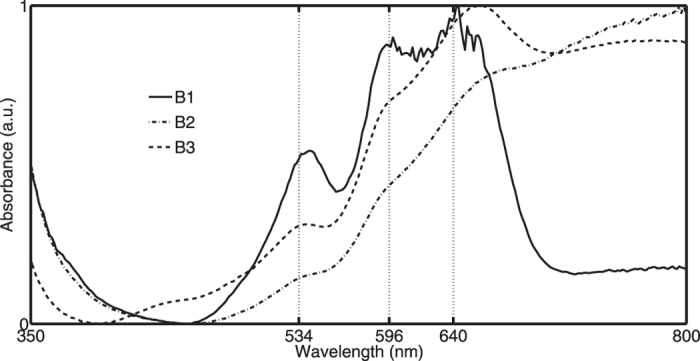
Spectral analysis of all blue fragments unveils three separate groups B1, B2 and B3 (the sample numbers of each group population are given in Supplementary Table 1). The first Co^2+^ absorption band is close to 534 nm favouring a soda-rich glass matrix.

The classification based on overall spectral shape of the spectra belonging to the blue panes leads to three groups B1–3 (Fig. 2). Maximum transmission occurs between 350–500 nm; the three groups differ in spectral position of the transmission maximum with averaged values of respectively 456.0 (±1.4), 459.6 (±1.7) and 393.0 (±1.4) nm. Analysis of the absorbance spectra indicate the presence of cobalt as colouring element. Cobalt is characterised by the presence of three successive absorption bands close to 535 nm, 596 nm and 640 nm attributed to the Jahn-Teller split transition A2 → T1(P) of Co^2+^ tetrahedral coordinated by four oxygen ions^25^. In all spectra of the blue fragments these bands are visible. Apparent is the common spectral position of the first cobalt absorption band of all blue fragments close to 534.2 nm (±1.6). Ceglia *et al.* correlates the spectral shift of this band to the composition of the historical glass^26^. Glasses with a calco-potassic composition have a band situated around 526.5 nm (±1.5 nm) shifting towards longer wavelengths (535 nm ±2 nm) in case of a soda based composition. These observations correspond with those made by Green and Hart^27^. Therefore, we conclude that the blue panes contain cobalt as colouring agent in a soda containing glass matrix and thus we can exclude a 14^th^–15^th^ century origin.

An important element for dating green pot-coloured panes is chromium; an element which typically distributes into a trivalent Cr^3+^ and a hexavalent Cr^6+^ state. The trivalent state is characterized by several absorption bands in the visible region which correspond to the *d-d* spin allowed ligand field transitions and results in an emerald green colour. The hexavalent state owes one strong charge transfer band in the ultraviolet part of the spectrum and imparts a lemon yellow colour. Scholars report data on the spectral positions of both ion states all referring to the pioneering work of Bamford, Weyl and Nath and Douglas^28^,^29^,^30^. The glass fragments used in these references are custom made glass fragments and include lithium, sodium and potash silicate glasses. In sodium silicate glasses the absorption peaks are found to be centred around 450 nm, 633 nm, 646 nm and 682 nm for Cr^3+^ and at around 365 nm for Cr^6+^. The presence of a sufficient amount of Cr^6+^ most often masks the Cr^3+^ absorption peak at around 450 nm. Unfortunately, the differences in spectral positions of the absorption peaks in different glass matrices are smaller than 5 nm. Therefore, inferences on the glass matrix based on the analysis of the spectral positions of the chromium absorption bands are useless. However, as it is generally believed that the exploitation of chromium as colouring element in glass industry only starts from the second half of the 19^th^ century onwards, we can focus our research to the identification of the presence/absence of the chromium signature. However, the fact that chromium could also enter the glass batch via the cobalt ores has to be taken into account.

**Figure 3:**
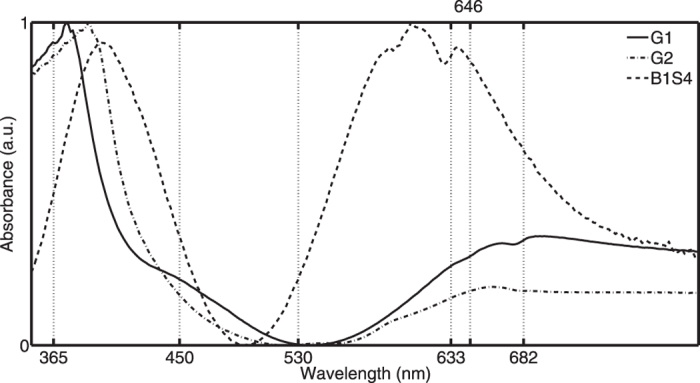
All green fragments could be classified in two groups (G1 and G2) based on spectral shape differences (the sample numbers of each group population are given in Supplementary Table 1). Cr^3+^ and Cr^6+^ optical fingerprints are observed in all green pot-coloured panes. The observed spectral positions of the chromium bands match with those of a reference sample B1S4 (chemical data is provided in Supplementary Table 2).

All the green panes show maximum transmission close to 530 nm (Fig. 3). Spectral analysis unveils two groups G1–2 due to a different spectral position of the transmission maximum respectively at 539.3 (±2.1) and 522.5 (±1.4) nm. The above mentioned Cr^3+^ bands are visible in all absorbance spectra. In addition, it appears that they contain a considerable amount of Cr^6+^. Accordingly, the corresponding absorption band around 365 nm is clearly visible influencing the strength of the Cr^3+^ absorption band at 450 nm. In Fig. 3 we demonstrate how the observed chromium absorption bands of pane N°54 coincide with those of a reference sample B1S4 for which the presence of chromium was proven via SEM-EDX analysis (Supplementary Table 2). The linear absorption coefficient of cobalt is large (approximately a factor of five for soda-silica-lime) compared to other metal oxides. This makes that the presence of trace levels of cobalt can easily be optically detected. Since we did not observe any cobalt absorption band in the recorded optical spectra we conclude that it is unlikely that the chromium oxides entered the batch as trace element originating from cobalt ores. We assume that the glasses were intentionally coloured with chromium. Therefore we conclude that the green pieces cannot be dated before the second half of the 19^th^ century.

**Figure 4:**
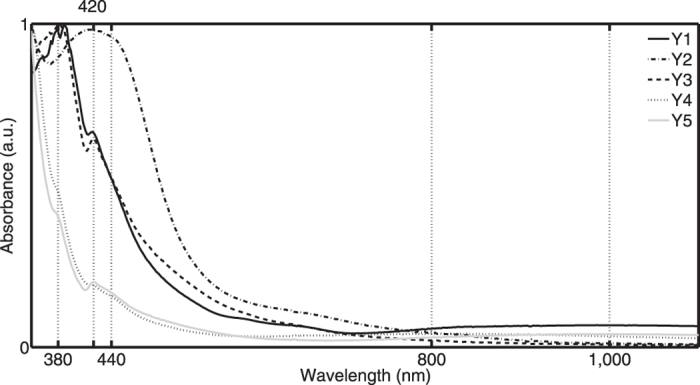
Yellow pot-coloured glasses are spectrally classified in five groups (Y1–Y5) reflecting differences in Fe^2+^/Fe^3+^ ratio and absence/presence of Co^2+^ and Cu^2+^ ions (the sample numbers of each group population are given in Supplementary Table 1).

Yellow glass is used for the border parts of both panels. The panes of panel N°55 are close to the yellow-white boundary of the horse-shoe colour diagram. Five separate spectral groups are recognized (Fig. 4). The first two groups are characterized by the presence of the main Fe^2+^ absorption peak and by weak Co^2+^ absorption bands. The main absorption band of Fe^2+^ is close to 1000 nm. Groups Y1–2 differentiate by their spectral position of the transmission maximum respectively at 711.9 (±3.1) nm and 697.5 (±1.4) nm. The spectral shape of the third group (Y3) indicates the presence of Fe^3+^ with absorption bands close to 380, 420 and 440 nm. These spectra are purer with gradual decreasing absorbance values starting from 450 nm onwards and converging to a constant level at a wavelength close to 1000 nm. Also the contour parts of panel N°55 are mainly coloured by the presence of Fe^3+^ ions. These fragments are classified in two groups (Y4–Y5) distinguished by the spectral position of the transmission maximum (605.4 ± 10.6 nm and 685.5 ± 1.4 nm). The presence of an additional band close to 800 nm for the fourth group (Y4) is probably caused by the presence of Cu^2+^ ions. Since no link has currently been uncovered between differences in Fe^2+^/Fe^3+^ ratio and the absence/presence of Co^2+^ and Cu^2+^ ions and the glasses’ chronological or geographical origin, we cannot draw any conclusion on the date of production of the yellow parts. Nevertheless, the authors decided to include the spectroscopic data to enable the immediate validation of this data in case future developments related to this issue are made by us or other research groups.

### Flashed glass

**Figure 5:**
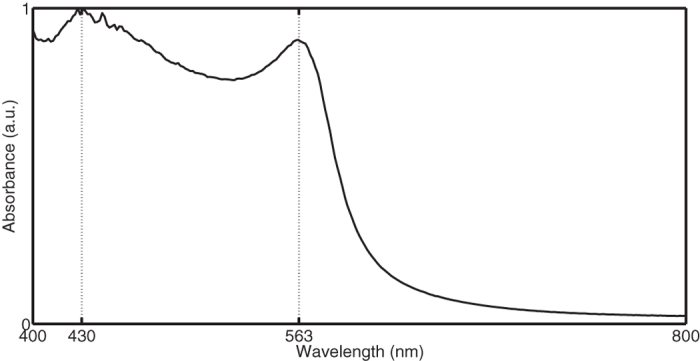
The flashed red glasses display the typical copper SPR signature with peaks at 430 and 563 nm.

Panel N°54 contains ten red panes. The characteristic copper surface plasmon resonance (SPR) peak, clearly visible in the measured spectra (Fig. 5), confirms the presence of Cu^0^ nanoparticles. The reduced glasses show a narrow peak at 563.1 (±0.8) nm generated by the colloidal dispersion of the metallic nanocrystals in the glass and a broader spectral contribution centred at 430 nm. The latter is usually correlated with isolated Cu^0^ atoms in the glass^31^. Although all ten samples have a quite similar spectral shape, some differences in peak intensity are observed. The peak at 430 nm is most distinct for fragment 54–44 and is weaker but still clearly observable for parts 54–13, 54–19, 54–20 and 54–40. It is absent for the remaining panes. The position of the SPR peak together with the slope of the absorbance curves between 400–500 nm points out the absence of europium ions^31^.

The colour uniformity observed by naked eye and with a magnifying glass indicates an even size distribution of the nanoparticles and favours a classification in the so-called ‘plaqué type’ structural group. The absence of the typical ‘wavy’ pattern due to the presence of red striae brought us to the conclusion that a sandwich structure is unlikely. We drew this conclusion based on the work described by Jerzy J. Kunicki-Goldfinger^32^ in which the macroscopic observation of the presence of a sandwich structure was confirmed via TEM examination. However in our case TEM examination was not possible as the museum stipulated solely fully non-destructive research. As a consequence no sampling, or any gentle material preparation was allowed.

The average cluster radii *R* of the embedded nanoparticles are calculated from the full-width half-maximum (FWHM) of the optical absorption peaks using an extrapolation of the formula provided by Manikandan^33^. The average particle radius of 11.0 (±0.7) nm agrees with the particle sizes described by Kunicki-Goldfinger^32^.

**Figure 6:**
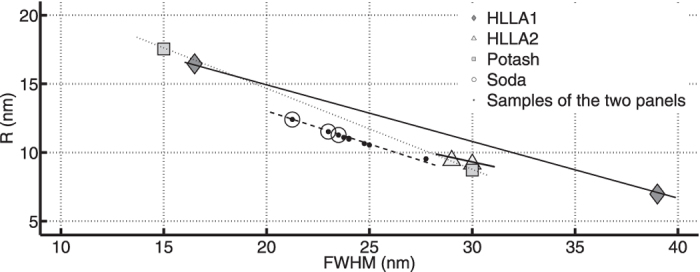
The FWHM-*R* correlation for the red flashed glasses is clearly visible. The slope of the trend-line depends on the glass matrix. All samples taken from the two panels fit to the soda-rich material.

The position and shape of the SPR band depends on the structure and distribution of the clusters as well as on the dielectric functions of the metal- and the glass matrix and the annealing temperature. From Manikandan’s research^33^ we know that in case of copper nanoparticles in soda lime glasses there is a blue shift of the SPR wavelength and a decrease in particle size (FWHM increases) with increasing annealing temperature. These findings were in agreement with the research of Kreibig and Vollmer^7^. In an attempt to gather more information on the production technique and period, we compared these values with the dispersion properties and quantum dot sizes of all other red copper fragments studied by our research group in the past decade. Despite the limited number of studied fragments some conclusions can be drawn when plotting the nanoparticles radii *R* as a function of the FWHM values (Fig. 6). The correlation between both parameters is clearly visible and highlighted by plotting trend lines. A difference in slope is observed for the potash, HLLA and soda glasses. We consider two groups of HLLA material (see Supplementary Table 2) classified mainly on differences in alkali metal concentration levels. All the samples taken from the two panels fit to the FWHM-*R* trend line of the soda rich material. Both groups also have matching SPR peak position values (Supplementary Table 1). However, the latter is not conclusive; the SPR values of the soda rich material are blue shifted compared to the HLLA material but have quantitative similar values as the potash fragments.

The spectroscopic properties exclude a pure potash rich as well as a pure soda rich glass composition and favours a mixed-alkali glass composition for the red pieces. This fact rules out a 14^th^ century origin which would favour a pure potash rich composition. However, the presence of potash ions rejects the assumption that we are dealing with modern panels fabricated during the industrial period which would imply a pure soda rich composition. Along with the blue and green fragments, we can conclude that also the red flashed parts cannot be dated to the 14^th^ century.

### Naturally-coloured panes

**Figure 7:**
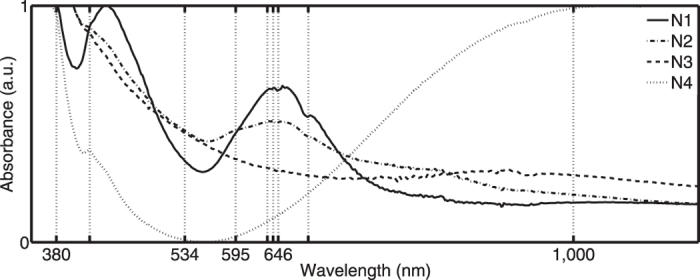
Naturally coloured panes contain chromium, cobalt and/or iron traces depending on the spectral group in which they are classified. The fragments are classified in four spectral groups N1–N4 (the sample numbers of each group population are given in Supplementary Table 1).

These fragments cover the centre part of the horse-shoe curve and can be classified in four spectral groups (Fig. 7). A first group P1, containing panes from both panels, is characterized by several absorption bands whose positions close to 381 nm, 446 nm, 638 nm, 644 nm and 686 nm indicate the presence of Fe^3+^ and Cr^3+^. The three fragments of panel N°55 correspond to the white glass decorated with silver yellow. The second group P2 contains white quarries both with and without silver stain. Also here we observe different spectral absorption bands. The discerned spectral positions at 380 nm, 418 nm, 594 nm, 641 nm and 684 nm designate the manifestation of chromium, cobalt and/or iron traces. The panes of the remaining two groups show a much cleaner profile with an almost lack of trace element bands. The plain fragments of the third group P3 have large absorbance values possibly caused by the presence of grisaille paint traces. The single fragment of the last group P4 displays the typical ferrous absorption band close to 1050 nm. The absorption band at 418 nm suggests the formation of an iron-manganese complex^34^. The fact that the naturally-coloured parts display a rather impure composition reflected by the presence of several trace elements, makes it very unlikely that the non-coloured parts are fabricated with an industrial controlled procedure since modern, industrial glasses are typically characterized by a very pure composition.

### Silver stain decorations

**Figure 8:**
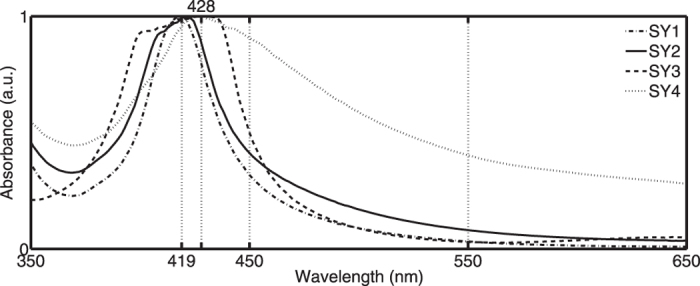
Spectral signature differences classify all stained fragments in four groups SY1–SY4 (the sample numbers of each group population are given in Supplementary Table 1). SY1–3 have an almost coincident absorption maximum close to 419 nm and differ in FWHM value. Panes of SY4 have red-shifted spectra with broad FWHM values tailing towards 450–550 nm.

Both panels contain several silver stained fragments. Study of the spectral signatures reveals four separate groups: SY1–4 (Fig. 8). All spectra of SY1–3 are characterized by an almost coincident spectral position of the absorption maximum close to 419.4 (±2.9) nm. The classification is based on a difference in spectral bandwidth ranging between 22.5–75 nm. In the case that the silver stain pieces were fabricated under equal firing temperatures, the appearing coincident absorption peak maxima values might indicate a similar firing temperature with the difference in FWHM pointing out a change in particle dimensions. Using Doyle’s formula^35^ the calculated corresponding average cluster radii *R* span 1.8–5.7 nm. Since almost the entire central motif is decorated with silver stain belonging to SY1–2, hypothetically it is plausible that both groups contain an equal glass type and reflect the genuine manufacturing of the panel. SY3 spectra demonstrate a clear second (weaker) blue shifted band between 380–450 nm. This band is already visible in the second group of panes; though at a much lower intensity. Mock *et al.*^36^ describes the development of a single band due to a formation of spherical particles while the appearance of two bands may originate from either a bi-modal distribution of nearly spherical particles or a distribution of particles with non-spherical symmetry. Currently the cause of the appearance of this second band remains an open question. Referring to the spectral similarities of the SY1–3 stained fragments a possible explanation might be that the double shifted peaks are caused by a higher density of particles^6^ and the groups simply correspond to the use of different concentrations of precursor mixture. The SY4 stained fragments have a deviant spectral shape. The peak maxima are red shifted near 428.2 (±2.6) nm and the broad band with FWHM value close to 67 nm has a tail towards the 450–550 nm region. This shape leads to an orange hue which might indicate the use of clays^35^ or the presence of a mixture of Ag and Cu in the paste composition^37^. The strong correlation in spectral shape of these spectra with the laboratory-made glass fragments reported in Delgado’s paper^37^, favours the second option.

**Figure 9:**
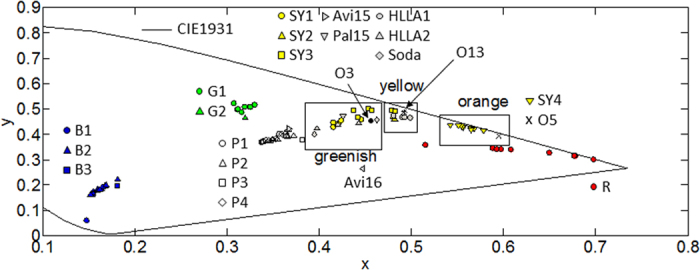
Colour values of all measured panes. All studied silver-yellow colours have a greenish, yellowish or orange hue on the CIE1931 colour diagram.

**Figure 10:**
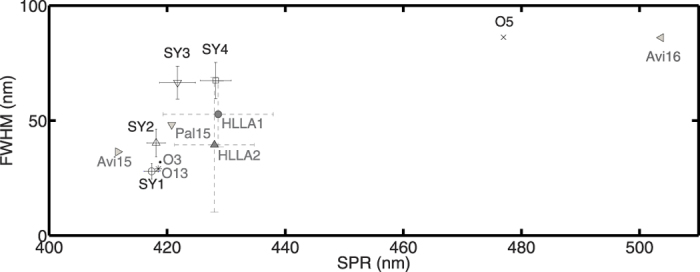
Standard deviations on the SPR and FWHM of the silver-stain material of the two panels of Koksijde are small compared to silver stained material originating from a 16^th^ century context (HLLA composition).

A variety of mainly fabrication and material related parameters determine the final colour. At present only a few authors have applied the silver stain process on custom-made glasses in order to unveil the fabrication techniques in medieval times. The limited amount of research at this stage makes a decision about fabrication conditions and corresponding period based on colour and spectral shapes difficult. However, since each fabrication condition (glass type, paint type and concentration, firing temperature and time) leads to a characteristic spectral fingerprint we compared the colour and spectral shapes of all SY1–4 stained glasses with those of ancient fragments found in literature. The glass compositions of these fragments include HLLA and mixed-alkali; two of the three major glass types used between 800–1800 AD. It concerns ten HLLA fragments from two Spanish (Avila & Palencia)^6^ (Avi15, Avi16 and Pal15) and one Belgian (Bruges)^38^ location together with three (O3, O5 and O13) mixed-alkali pieces from Tomar (Portugal)^37^. The HLLA samples from Bruges are further classified in HLLA1 and HLLA2 following their differences in alkali metal concentration levels. Chemical data is given in Supplementary Table 2. Three observations are made. (1) Apart from the two samples originating from Avila, the calculated colour values group all fragments in three separate classes characterized by a greenish-yellow, yellow or orange colour (Fig. 9). (2) Secondly, it is perceived that the colour values as well as the entire spectral shape of the three mixed-alkali fragments correspond pretty well with the stained fragments of the two studied panels. (3) Finally, it is concluded that the standard deviations on the spectral properties (error flags in Fig. 10) of the latter are much smaller compared to the two HLLA groups of post-medieval material. This points out a better controlled fabrication process potentially implying a more recent production.

Several types of silver compounds can be used affecting the final appearance. Jembrih-Simbürger^39^ has proven that the silver compound type affects the distribution of the particles resulting in uniform or speckled layers. Since none of the stained panes show a speckled surface, the silver salt was most probably AgNO_3_. In addition, comparison of the spectral data with the samples described by Pérez-Villar^35^ did not unveil any similarity favouring the idea of the absence of clay in the silver mixture. Jembrih-Simburger^39^ also reports on the relation between colour intensity, applied silver salt and firing temperature. More in particular the silver compounds are divided in three groups: (1) silver compounds resulting in an intense colour at low firing temperatures such as AgNO_3_ and Ag_2_SO_4_; (2) compounds resulting in a paler colour at low firing temperatures such as AgCl and Ag_3_PO_4_; and (3) compounds that hardly result in any colour such as Ag_2_O. The information given in this paper together with the analysed colours and defined silver salt predicts a low firing temperature for the two panels. As the staining process takes place at a temperature close to the glass transition temperature, this implies a mixed alkali or a soda rich composition; a conclusion which is based on the glass transition temperatures reported in literature (533 °C mixed alkali glass^35^; 564 °C soda lime glass; 653 °C high lime low alkali glass^6^).

We conclude that these data give an additional prove of non-authenticity; the presence of a Cu-Ag mixture in the glass paint suggests that the pieces were not produced before the 16^th^ century. Three factors strengthen the assumption of a mixed-alkali composition suggesting a fabrication date between the end of the 17^th^ century and the start of the industrial glass production from the mid 19^th^ century onwards: (1) The fabrication process of the silver stained parts of the two panels seems to be more controlled compared to earlier dated glasses with a HLLA composition (end 15^th^–end 17^th^ century); (2) The similarity in spectral properties between the stained fragments of the two panels and historic well-dated fragments with a mixed-alkali composition and (3) Staining process temperatures which are close to the glass transition temperature of mixed-alkali glasses. Finally, there is also an additional prove for a non-industrial fabrication being the absence of selenium, europium and cadmium nanoparticles commonly used in 20^th^ century red stained glasses.

### Dating criteria discussion

Several identified spectral groups contain fragments from both panels. Therefore, it is most likely to date both panels in a similar period.

The spectroscopic research outcome excludes an early 14^th^ century origin because of three main reasons: the absence of a pure potash rich glass composition, the presence of colouring elements or fabrication techniques only used in later periods and the evidence of a rather controlled fabrication process leading to quite uniform spectral shapes with small deviations within one group.

The spectral position of the cobalt absorption bands of all blue fragments and the copper nanoparticle signatures of all red flashed glasses indicate a soda rich nature.

The presence of chromium dates all green pieces in a recent period. Kunicki-Goldfinger *et al.*^32^ described a chronological occurrence of flashed red plaqué glasses from the late fourteenth century onwards. Although this concerned a study of British and French material, a geographical expansion is not illusive. Twenty-five percent of the silver stained fragments seems to be painted with a Cu-Ag mixture. Following Molina^6^ this would mean that these pieces were not produced before the sixteenth century.

The spectral properties indicate that not all red parts were placed at the same time or that they originate from different plates. Though, their close position on one straight line in the FWHM-*R* scatter plot denotes similar fabrication conditions.

Seventy-five percent of all silver stained fragments have almost coincident positions of the peak maxima indicating a similar firing temperature. The tail spreading differences between the three subgroups points to a change in precursor mixture concentration. In addition, it can be concluded that the standard deviations for all four silver stained groups of Koksijde are small compared to the two HLLA groups of post-medieval material from Bruges. This might point out a better controlled fabrication process for the Koksijde material.

Several observations hint a mixed-alkali glass composition. For the silver stained fragments the strong correlation in spectral shape with the mixed-alkali samples from Tomar is distinct.

If we indeed assume that we are dealing with AgNO_3_ silver salts for the Koksijde material and taking into account that the staining process takes place at a temperature close to the glass transition temperature, this would imply a mixed alkali or a soda rich composition. The spectral similarities of the Ag-Cu silver stained fragments with the custom-made fragments of Delgado unveils the presence of potash atoms. Since earlier conclusions also proofed a presence of soda atoms, a soda-potash mixture is plausible. In addition the absence of red colouring nanoparticles such as selenium, europium and cadmium commonly used in the early 20^th^ century^31^,^40^ voids a pure industrial-based soda rich glass composition. Yet another fact that strengthens this allegation is the rather impure plain glass composition. The presence of trace elements possibly introduced via a wood ash based flux almost excludes a potential glass fabrication of these fragments within a highly controlled industrial environment.

Although the precise boundaries of production date still need some refinement, it is generally accepted that mixed-alkali glasses can be roughly dated between the end of the 17^th^ century until the end of the 19^th^ century. For this particular case we make the hypothesis that the two panels are most probably produced in the second half of the 19^th^ century. The latter is based on two specific observations: (1) the presence of chromium atoms (from second half of 19^th^ century onwards) and (2) the absence of red colouring selenium, europium and/or cadmium nanoparticles (from the early 20^th^ century onwards).

## Methods

### Spectroscopic analysis

Since both panels form part of the museum’s permanent exhibition, all analysis steps had to be finalized within one week. For this research the panels could not be dismantled and none of the glass fragments were taken out of the lead matrix. In order to enable good measuring both panels were temporarily disembodied from their non-transparent perspex carrier. Afterwards we measured the transmission spectra of all mentioned parts. Finally the panels were replaced onto the perspex carrier.

For each defined location we recorded the transmittance spectrum T(λ) between 300–1500 nm; i.e. the spectral region where most absorption bands are located. Afterwards we calculated the absorbance spectra using the formula A(λ) = −*log*_10_T(λ). A spectral broadband light source (Avalight-HAL + DHSBAL; Avantes) illuminated the sample; an optical spectrum analyser (AvaSpec-3648, AvaSpec-256-NIR1.7; Avantes) was used to measure the transmitted intensity as a function of the wavelength. The analysed area has a circular surface area close to 10 mm^2^. Spectra are recorded with a spectral resolution of 1.4 nm.

For the decorations two measuring points were taken if possible, considering both the glass and the silver stain.

The external properties of the panels only allow the application of a so-called ‘relative’ measurement configuration. It uses an optical fibre with a large acceptance angle (NA = 0.22) to capture most of the transmitted light. This is in contrast with an ‘absolute’ configuration where all the transmitted light is guided towards the spectrum analyser using an integrating sphere. The consequence is that we are not able to draw quantitative conclusions about the chemical composition. This was anyway not required for this study.

### Calculation of the average cluster radius *R*

The average cluster radii R of the embedded nanoparticles are calculated using an extrapolation of equation (1) which is the formula provided by Manikandan^33^:


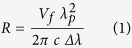


where V_f_ is the Fermi velocity of the electrons in bulk metal (copper = 1.57 × 10^8^ cm/s; silver = 1.39 × 10^8^ cm/s), Δλ is the full-width at half maximum of the absorption band and λp is the characteristic wavelength at which surface plasmon resonance (SPR) occurs.

### Colour analysis

The colour values which we represent on the widely used CIE*xy* 1931 colour diagram are derived from the measured transmission spectra. In a first step this spectrum is multiplied with the colour matching functions and the spectrum of the source under which we observe the object. We calculated the colour for an equal energy illumination. Next, integration of the three obtained spectra between 380–780 nm (i.e. the spectral region for which the human eye is colour sensitive) leads to three values *X*, *Y* and *Z.* These values result after normalization in the colour values *x* and *y* which are represented on the horse-shoe curve. All colours that are physically perceivable by the human eye can be located inside the closed figure on this graph. The curved line is the spectral locus and represents all monochromatic colours.

## Conclusion

The work described in this paper has been a model of collaboration between art historians and optical engineers in the context of cultural heritage research. Profound re-examining of the macroscopic material properties and stylistic comparison of the two stained glass panels questioned their authenticity. In addition to the macroscopic clues, spectroscopic analysis has now confirmed the incorrect dating of both panels to the 14^th^ century. The integrated research offers persuasive evidence that the production date of both panels is far more likely to be placed in the second half of the 19^th^ century. As the abbey was abandoned in 1578 and further dismantled in 1625, this conclusion is of considerable significance indicating that neither of the two panels can have come from the Abbey of the Holy Mary of the Dunes. For the museum ‘Ten Duinen 1138’, this new insight will imply a redesign of the presentation of both panels, since they can no longer be presented as authentic pieces from the former Cistercian Abbey of the Dunes.

This paper therefore demonstrates the important role optical spectroscopy can play in the enigma of authenticity questions of stained glass.

## Additional Information

**How to cite this article**: Meulebroeck, W. *et al.* Authenticity screening of stained glass windows using optical spectroscopy. *Sci. Rep.* **6**, 37726; doi: 10.1038/srep37726 (2016).

**Publisher's note:** Springer Nature remains neutral with regard to jurisdictional claims in published maps and institutional affiliations.

## Supplementary Material

Supplementary Information
