# Supplementary material for: Authenticity screening of stained glass windows using optical spectroscopy
Source: Sci Rep. 2016 Nov 24;6:37726. doi: 10.1038/srep37726 (PMC5121600; doi:10.1038/srep37726)
Supplement: Supplementary Information [file srep37726-s1.pdf]

Authenticity screening of stained glass windows using optical spectroscopy

Wendy Meulebroeck[1,*,+], Hilde Wouters[1,2,+], Karin Nys[2], and Hugo Thienpont[1]

1 Vrije Universiteit Brussel, Applied Physics and Photonics Department, Brussels Photonics Team B-PHOT, Brussels, 1050, Belgium

2 Vrije Universiteit Brussel, Department of Art Studies and Archaeology, Brussels, 1050, Belgium

* wmeulebr@vub.ac.be

+ these authors contributed equally to this work

Supplementary Table 1. Spectral features of all measured panes.

Pot-coloured / blue

Sample	*x*	*y*	Colour	Position Co^{2+} 1st absorption band (±1.4 nm)	Position transmission maximum (±1.4 nm)	Additional bands (±1.4 nm)
**B1**						
54-5	0.15	0.06	purplish blue	534.0	456.0	* strong IR Co^{2+} bands (1225.0, 1266.0, 1483.5)<br>* Cu^{2+} (838.0)
**B2**						
55-2	0.15	0.17	blue	535.5	463.5	-
55-4A	0.16	0.18	blue	532.5	459.0	-
55-6	0.16	0.18	blue	531.0	460.5	-
55-8	0.17	0.19	blue	532.5	459.0	-
55-11	0.16	0.18	blue	537.0	460.5	-
55-12	0.15	0.16	blue	535.5	460.5	-
55-16	0.16	0.18	blue	534.0	457.5	-
55-18	0.16	0.17	blue	534.0	460.5	-
55-22	0.17	0.19	blue	535.5	460.5	-
55-24	0.15	0.16	blue	532.5	459.0	-

55-27	0.17	0.20	blue	534.0	457.5	-
55-29	0.16	0.18	blue	535.5	459.0	-
55-32	0.18	0.22	blue	535.5	457.5	-
**B3**						
55-4B (restored)	0.18	0.20	blue	534.0	393.0	weak band around 440.0

Pot-coloured / green

Sample	*x*	*y*	Colour	Cr^{3+} absorption bands present?	Cr^{6+} absorption bands present?	Position transmission maximum (±1.4 nm)
**G1**						
54-11	0.33	0.51	yellowish-green	yes	yes	541.5
54-16	0.33	0.51	yellowish-green	yes	yes	541.5
54-23	0.31	0.50	yellowish-green	yes	yes	537.0
54-26	0.33	0.52	yellowish-green	yes	yes	541.5
54-31A	0.31	0.50	yellowish-green	yes	yes	537.0
54-37	0.32	0.51	yellowish-green	yes	yes	540.0
54-47	0.32	0.49	yellowish-green	yes	yes	538.5
54-48	0.31	0.52	yellowish-green	yes	yes	537.0
**G2**						
54-31B (restored)	0.32	0.46	yellowish-green	yes	yes	522.5 & 553.5

Pot-coloured / yellow

Sample	*x*	*y*	Colour	Observed ions & absorption positions (±1.4 nm)	Position transmission maximum (±1.4 nm)
**Y1**					
54-1	0.54	0.45	yellow	Fe^{2+} (993.0) Co^{2+} (640.5, 648.0)	712.5
54-9A	0.49	0.46	yellow	Fe^{2+} (994.5) Co^{2+} (639.0, 645.0)	717.0
54-18	0.49	0.46	yellow	Fe^{2+} (985.5) Co^{2+} (642.0, 649.5)	715.5
54-21	0.52	0.46	yellow	Fe^{2+} (1024.5) Co^{2+} (640.5, 646.5)	709.5
54-27	0.48	0.46	yellow	Fe^{2+} (1087.5) Co^{2+} (639.0, 645.0, 648.0)	709.5
54-30	0.48	0.46	yellow	Fe^{2+} (1053.0) Co^{2+} (643.5, 648.0, 651.0)	708.0
54-38	0.48	0.46	yellow	Fe^{2+} (1053.0) Co^{2+} (643.5, 652.5)	712.5
54-49	0.49	0.46	yellow	Fe^{2+} (1030.5) Co^{2+} (646.5, 651.0)	711.0
**Y2**					

54-46	0.49	0.46	yellow	Fe^{2+} (1053.0) Co^{2+} (639.0, 643.5, 646.5)		697.5
**Y3**						
54-9B (restored)	0.49	0.46	yellow	Fe^{3+} (417.0)		convergence (near-infrared region)
54-22 (restored)	0.51	0.44	yellow	Fe^{3+} (381.0, 420.0)		convergence (near-infrared region)
**Y4**						
55-1	0.38	0.39	yellow / white	Fe^{3+} (417.0) Cu^{2+} (822.0)		606.0
55-9	0.42	0.43	yellow / white	Fe^{3+} (415.5) Cu^{2+} (829.5)		613.5
55-13	0.41	0.43	yellow / white	Fe^{3+} (415.5) Cu^{2+} (853.5)		601.5
55-14	0.41	0.44	yellow / white	Fe^{3+} (417.0) Cu^{2+} (825.0)		600.0
55-15	0.42	0.43	yellow / white	Fe^{3+} (417.0) Cu^{2+} (823.5)		621.0
55-19	0.38	0.40	yellow / white	Fe^{3+} (417.0) Cu^{2+} (826.5)		610.5
55-20	0.39	0.40	yellow / white	Fe^{3+} (417.0) Cu^{2+} (820.5)		615.0
55-21	0.40	0.42	yellow / white	Fe^{3+} (417.0) Cu^{2+} (823.5)		609.0
55-25	0.39	0.41	yellow / white	Fe^{3+} (417.0) Cu^{2+} (825.0)		607.5
55-26	0.40	0.42	yellow / white	Fe^{3+} (417.0) Cu^{2+} (837.0)		597.0
55-30	0.39	0.41	yellow / white	Fe^{3+} (417.0) Cu^{2+} (810.0)		613.5
55-31	0.39	0.41	yellow / white	Fe^{3+} (417.0) Cu^{2+} (823.5)		595.5
55-33	0.40	0.42	yellow / white	Fe^{3+} (417.0) Cu^{2+} (850.5)		580.5
**Y5**						
55-10	0.38	0.38	yellow / white	Fe^{3+} (420.0)		685.5

Flashed / red

Sample	*x*	*y*	Colour	SPR peak position & FWHM (nm)	Average particle radius *R* (nm)	Strength band linked to presence isolated Cu^0 atoms (~ 430 nm)
**R Koksijde**						
54-10	0.59	0.35	reddish-orange	562.5 & 24.0	11.0	absent
54-13	0.65	0.33	reddish-orange	564.0 & 24.0	11.0	weak
54-14	0.52	0.36	reddish-orange	562.5 & 24.0	11.0	absent
54-17	0.60	0.34	reddish-orange	562.5 & 21.3	12.4	absent
54-19	0.68	0.32	reddish-orange	562.5 & 24.8	10.6	weak
54-20	0.68	0.32	reddish-orange	562.5 & 25.0	10.5	weak
54-32	0.61	0.34	reddish-orange	564.0 & 23.5	11.3	absent
54-36	0.59	0.34	reddish-orange	564.0 & 23.0	11.5	absent

54-40	0.68	0.32	reddish-orange	564.0 & 27.8	9.5	weak
54-44	0.70	0.30	reddish-orange	562.5 & 23.8	11.1	strong
**Other sites**						
Dendermonde (potash)						
1	0.69	0.31	reddish-orange	560.5 & 30.0	8.7	strong
2	0.69	0.31	reddish-orange	562.0 & 15.0	17.5	strong
Bruges (HLLA)						
B6s36	0.71	0.29	reddish-orange	571.0 & 16.5	16.5	strong
B6s37	0.70	0.30	reddish-orange	571.5 & 39.0	7.0	strong
A6s50	0.70	0.29	reddish-orange	573.0 & 29.0	9.4	weak
C6s54	0.70	0.30	reddish-orange	574.5 & 30.0	9.2	strong
Bruges (soda)						
A3s2	0.70	0.30	reddish-orange	564.0 & 23.5	11.3	strong
Glashütte Lamberts (soda)						
1001U Hell	0.60	0.34	reddish-orange	564.0 & 23.0	11.5	absent
1001U Mittel	0.69	0.31	reddish-orange	562.5 & 21.3	12.4	strong

Naturally coloured

Sample	*x*	*y*	Colour	Observed ions & absorption positions (±1.4 nm)	Position transmission maximum (±1.4 nm)
**N1**					
54-41	0.36	0.40	white	Fe^{3+} (379.5) Cr^{3+} (439.5, 636.0, 657.0, 684.0)	405.0 & 555.0
54-43	0.36	0.40	white	Fe^{3+} (375.0) Cr^{3+} (441.0, 636.0, 655.5, 684.0)	405.0 & 556.5
55-3	0.34	0.37	white	Fe^{3+} (381.0) Cr^{3+} (445.5, 637.5, 655.5, 685.5)	397.5 & 547.5
55-5	0.34	0.37	white	Fe^{3+} (379.5) Cr^{3+} (439.5, 639.0, 657.0, 687.0)	397.5 & 547.5
55-7	0.34	0.38	white	Fe^{3+} (378.0) Cr^{3+} (444.0, 637.5, 657.0, 684.0)	397.5 & 550.5
**N2**					
54-3	0.37	0.39	white	Fe^{3+} (378.0, 417.0) Co^{2+} (597.0, 649.5) Cr^{3+} (636.0, 655.5, 684.0)	561.0
54-7	0.37	0.39	white	Fe^{3+} (375.0, 417.0) Co^{2+} (597.0, 651.0) Cr^{3+} (636.0, 655.5, 684.0)	565.5
54-12	0.36	0.39	white	Fe^{3+} (378.0, 417.0) Co^{2+} (595.5, 649.5) Cr^{3+} (637.5, 655.5, 685.5)	559.5
54-15	0.36	0.38	white	Fe^{3+} (378.0, 417.0) Co^{2+} (597.0, 649.5) Cr^{3+} (637.5, 655.5, 685.5)	559.5

54-28	0.34	0.37	white	Fe^{3+} (378.0, 420.0) Co^{2+} (595.5, 649.5) Cr^{3+} (639.0, 655.5, 685.5)	556.5
54-29	0.34	0.37	white	Fe^{3+} (375.0, 420.0) Co^{2+} (600.0, 649.5) Cr^{3+} (636.0, 655.5, 684.0)	553.5
54-33	0.36	0.41	white	Fe^{3+} (378.0, 418.5) Co^{2+} (597.0, 649.5) Cr^{3+} (637.5, 654.0, 684.0)	565.5
54-35	0.37	0.42	white	Fe^{3+} (378.0, 418.5) Co^{2+} (595.5, 649.5) Cr^{3+} (636.0, 655.5, 684.0)	559.5
54-39	0.35	0.37	white	Fe^{3+} (375.0, 418.5) Co^{2+} (598.5, 649.5) Cr^{3+} (636.0, 655.5, 682.5)	558.0
54-45	0.35	0.38	white	Fe^{3+} (376.5, 417.0) Co^{2+} (597.0, 649.5) Cr^{3+} (636.0, 655.5, 685.5)	558.0
54-50	0.35	0.37	white	Fe^{3+} (376.5, 417.0) Co^{2+} (597.0, 649.5) Cr^{3+} (636.0, 655.5, 684.0)	559.5
54-52	0.35	0.38	white	Fe^{3+} (378.0, 417.0) Co^{2+}(595.5, 649.5) Cr^{3+}(637.5, 655.5, 684.0)	558.0
**N3**					
54-4	0.38	0.38	white	Fe^{3+} (375.0, 382.5, 420.0)	730.5
54-6	0.38	0.38	white	Fe^{3+} (379.5, 418.5)	727.5
**N4**					
55-17	0.35	0.38	white	Fe^{2+} (1066.5) Fe-Mn complex (418.5)	555.0

Silver yellow

Sample	*x*	*y*	Colour	SPR peak position & FWHM (±1.5 nm)	Average particle radius *R* (nm)	2nd peak present between 380-450 nm in absorbance spectrum?
**SY1**						
54-4	0.42	0.44	greenish-yellow	418.5 & 27.0	4.8	no
54-6	0.42	0.43	greenish-yellow	415.5 & 22.5	5.7	no
54-34	0.45	0.46	greenish-yellow	418.8 & 31.0	4.2	no
54-42	0.42	0.45	greenish-yellow	417.0 & 29.0	4.4	no
54-51	0.42	0.45	greenish-yellow	417.0 & 30.0	4.3	no
**SY2**						
54-2	0.48	0.46	yellow	417.0 & 36.0	3.6	yes (weak band)
54-8	0.48	0.47	yellow	419.3 & 44.5	2.9	yes (weak band)
**SY3**						

54-25	0.44	0.47	greenish-yellow	420.0 & 64.5	2.0	yes (stronger band)
55-3	0.44	0.49	greenish-yellow	417.8 & 57.5	2.2	yes (stronger band)
55-5	0.48	0.49	greenish-yellow	424.5 & 75.0	1.8	yes (stronger band)
55-7	0.45	0.50	greenish-yellow	422.0 & 64.0	2.1	yes (stronger band)
55-23	0.46	0.49	greenish-yellow	419.8 & 63.5	2.0	yes (stronger band)
55-28	0.48	0.49	greenish-yellow	424.8 & 72.5	1.8	yes (stronger band)
**SY4**						
54-12	0.56	0.43	orange	424.5 & 54.0	2.5	no
54-15	0.56	0.43	orange	427.5 & 54.5	2.5	no
54-24	0.55	0.44	orange	428.0 & 74.0	1.8	no
54-28	0.54	0.44	orange	427.5 & 75.0	1.8	no
54-29	0.55	0.44	orange	430.5 & 70.5	1.9	no
54-39	0.58	0.41	orange	429.0 & 67.0	2.0	no
54-45	0.56	0.42	orange	427.5 & 73.5	1.8	no
54-50	0.57	0.42	orange	433.5 & 69.0	2.0	no
54-52	0.56	0.42	orange	426.0 & 69.0	1.9	no
**Other sites**						
Covento de cristo (Tomar, Portugal) – mixed alkali						
O3	0.46	0.45	greenish-yellow	418.8 & 31.9	4.1	no
O5	0.59	0.39	orange	477.0 & 86.2	1.9	yes (weak band)
O13	0.49	0.49	yellow	418.5 & 29.0	4.5	no
Avila and Palencia (Spain) - HLLA						
Avi15	0.37	0.42	yellow-green	411.7 & 36.4	3.4	no
Avi16	0.45	0.26	purplish-pink	503.7 & 86.0	2.2	yes (weak band)
Pal15	0.43	0.47	greenish-yellow	420.8 & 48.1	2.7	no
Bruges (HLLA1)						
A6s3	0.44	0.44	yellow	435.0 & 73.3	1.9	no
A6s7	0.42	0.44	yellow	427.5 & 23.0	5.9	yes (weak band)
A6s27	0.49	0.47	yellow	438.0 & 37.0	3.8	yes (weak band)
B1s15	0.40	0.42	yellow	421.5 & 22.0	6.0	yes (weak band)
B4s26	0.48	0.47	yellow	423.0 & 47.5	2.8	no
C3s37	0.49	0.47	yellow	418.5 & 71.0	1.8	no
C6s6	0.50	0.47	yellow	435.0 & 55.5	2.5	no
Bruges (HLLA2)						
A3s5	0.39	0.40	white	417.0 & 16.0	8.0	yes (weak band)

C3s39	0.46	0.46	yellow	423.0 & 36.5	3.6	no

Supplementary Table2. SEM-EDX analysis

Pot-coloured / green

Sample	SiO_2	Al_2O_3	Fe_2O_3	Cu_2O	Cr_2O_3	TiO_2	MnO	Na_2O	K_2O	MgO	CaO	Cl	SO_3	P_2O_5
Bruges														
B1s4	70.6	1.6	0.1	0.3	0.7	n.d	n.d	14.4	0.9	n.d	10.3	0.8	0.4	n.d

Flashed / red

Sample	SiO_2	Al_2O_3	Fe_2O_3	Cu_2O	Cr_2O_3	TiO_2	MnO	Na_2O	K_2O	MgO	CaO	Cl	SO_3	P_2O_5
Dendermonde (potash)														
1	-	-	-	-	-	-	-	-	-	-	-	-	-	-
2	51.7	2.3	0.6	n.d	n.d	0.5	1.5	1.2	17.7	4.8	16.6	0.4	n.d	2.9
Bruges														
HLLA1														
B6s36	59.6	3.3	0.6	n.d	n.d	0.3	0.6	2.7	3.7	3.5	21.5	0.7	0.3	3.1
B6s37	58.5	3.5	0.7	n.d	n.d	0.4	0.6	2.6	4.4	3.1	22.3	0.6	0.2	3.1
HLLA2														
A6s50	60.5	3.7	0.6	n.d	n.d	0.6	1.0	1.6	5.7	2.9	20.0	0.4	0.5	2.6
C6s54	60.8	3.8	0.6	n.d	n.d	0.6	0.8	1.6	5.7	2.9	20.1	0.4	0.4	2.5
Bruges (soda)														
A3s2	58.6	3.7	0.5	n.d	n.d	0.2	0.6	2.6	4.6	3.2	22.0	0.6	0.2	3.3
Glashütte Lamberts (soda)														
1001U Hell	-	-	-	-	-	-	-	-	-	-	-	-	-	-
1001U Mittel	-	-	-	-	-	-	-	-	-	-	-	-	-	-

Silver yellow

Sample	SiO_2	Al_2O_3	Fe_2O_3	Cu_2O	Cr_2O_3	TiO_2	MnO	Na_2O	K_2O	MgO	CaO	Cl	SO_3	P_2O_5
Covento de cristo (Tomar, Portugal) – mixed alkali														
O3	[Delgado	2011]												
O5	[Delgado	2011]												
O13	[Delgado	2011]												
Avila and Palencia (Spain) - HLLA														

Avi15	[Molina	2013]												
Avi16	[Molina	2013]												
Pal15	[Molina	2013]												
Bruges (HLLA)														
A6s3	59.6	4.0	0.8	n.d	n.d	0.5	0.6	1.5	5.6	2.8	21.1	0.4	0.3	2.8
A6s7	60.5	4.2	0.7	n.d	n.d	0.3	0.5	1.6	5.4	2.9	20.3	0.4	0.4	2.8
A6s27	60.1	4.1	0.8	n.d	n.d	0.4	0.6	1.6	5.6	2.9	20.4	0.4	0.3	2.8
B1s15	60.4	4.3	0.8	n.d	n.d	0.4	0.5	1.8	5.3	3.0	19.9	0.5	0.2	2.8
B4s26	58.7	4.3	0.6	n.d	n.d	0.5	0.6	1.8	5.8	2.8	21.4	0.5	0.3	2.9
C3s37	59.0	4.1	0.6	n.d	n.d	0.4	0.7	2.0	5.8	2.7	21.2	0.5	0.2	2.7
C6s6	59.8	4.1	0.8	n.d	n.d	0.4	0.6	1.7	5.6	2.9	20.7	0.4	0.2	2.8
